# Optimization of guanosine-based hydrogels with boric acid derivatives for enhanced long-term stability and cell survival

**DOI:** 10.3389/fbioe.2023.1147943

**Published:** 2023-03-20

**Authors:** Maria Merino-Gómez, Maria Godoy-Gallardo, Mathias Wendner, Miguel A. Mateos-Timoneda, F. Javier Gil, Roman A. Perez

**Affiliations:** ^1^ Bioengineering Institute of Technology (BIT), Faculty of Medicine and Health Sciences, International University of Catalonia (UIC), Sant Cugat del Vallès, Spain; ^2^ Department of Dentistry, Faculty of Dentistry, International University of Catalonia (UIC), Sant Cugat del Vallès, Spain

**Keywords:** guanosine-based hydrogels, boric acid derivatives, nucleoside, 3D printing, printable hydrogels

## Abstract

Tissue defects can lead to serious health problems and often require grafts or transplants to repair damaged soft tissues. However, these procedures can be complex and may not always be feasible due to a lack of available tissue. Hydrogels have shown potential as a replacement for tissue grafts due to their ability to support cell survival and encapsulate biomolecules such as growth factors. In particular, guanosine-based hydrogels have been explored as a potential solution, but they often exhibit limited stability which hampers their use in the biofabrication of complex grafts. To address this issue, we explored the use of borate ester chemistry and more complex boric acid derivatives to improve the stability and properties of guanosine-based hydrogels. We hypothesized that the aromatic rings in these derivatives would enhance the stability and printability of the hydrogels through added π-π stack interactions. After optimization, 13 compositions containing either 2-naphthylboronic acid or boric acid were selected. Morphology studies shows a well-defined nanofibrilar structure with good printable properties (thixotropic behaviour, print fidelity and printability). Moreover, the pH of all tested hydrogels was within the range suitable for cell viability (7.4–8.3). Nevertheless, only the boric acid-based formulations were stable for at least 7 days. Thus, our results clearly demonstrated that the presence of additional aromatic rings did actually impair the hydrogel properties. We speculate that this is due to steric hindrance caused by adjacent groups, which disrupt the correct orientation of the aromatic groups required for effective π-π stack interactions of the guanosine building block. Despite this drawback, the developed guanosine-boric acid hydrogel exhibited good thixotropic properties and was able to support cell survival, proliferation, and migration. For instance, SaOS-2 cells planted on these printed structures readily migrated into the hydrogel and showed nearly 100% cell viability after 7 days. In conclusion, our findings highlight the potential of guanosine-boric acid hydrogels as tissue engineering scaffolds that can be readily enhanced with living cells and bioactive molecules. Thus, our work represents a significant advancement towards the development of functionalized guanosine-based hydrogels.

## 1 Introduction

Hydrogels have gained increasing popularity as biomaterials in recent years for a wide range of applications, including drug delivery, cell culture, tissue engineering, and three dimensional (3D) printing (Drury and Mooney, 2003; Du et al., 2015; Thakur et al., 2019).

However, synthetic scaffolds aimed for tissue engineering applications still do not match the structural and functional complexity of natural human tissues, which contain a wide variety of cells and biomolecules. To address this, scaffold systems must be developed that are both adaptable and capable of incorporating living cells and bioactive molecules. In recent years, supramolecular hydrogels have received increasing attention due to their tuneable and reversible character based on small building blocks that link together to form complex networks. Thus, these materials are able to adapt to changing environmental conditions (Li et al., 2020) and can self-heal after experiencing damage (Godoy-Gallardo et al., 2023). Of all the types of supramolecular hydrogels studied that have these properties [e.g., fatty acids (Nuthanakanti and Srivatsan, 2020), sugars (Goel et al., 2021), cholesterol (Zhang et al., 2021), amino acids (Xie et al., 2020), peptides (Falcone et al., 2020)] and silk fibroin (Yao et al., 2022; Zou et al., 2022), nucleoside-based hydrogels appear most promising for tissue engineering applications. They have i) a shear modulus and thixotropic properties that make them highly suitable for extrusion-based 3D bioprinting, they can ii) hold living cells and bioactive molecules and support the survival of the entrapped cells, their iii) nanofibrillar and dynamic matrix mimics and stimulate the extracellular matrix while their iv) combination of elastic and fluid-like microviscosity properties is ideal for cell proliferation, migration, and differentiation. Finally, their v) chemistry is straightforward and simple.

**SCHEME 1:**
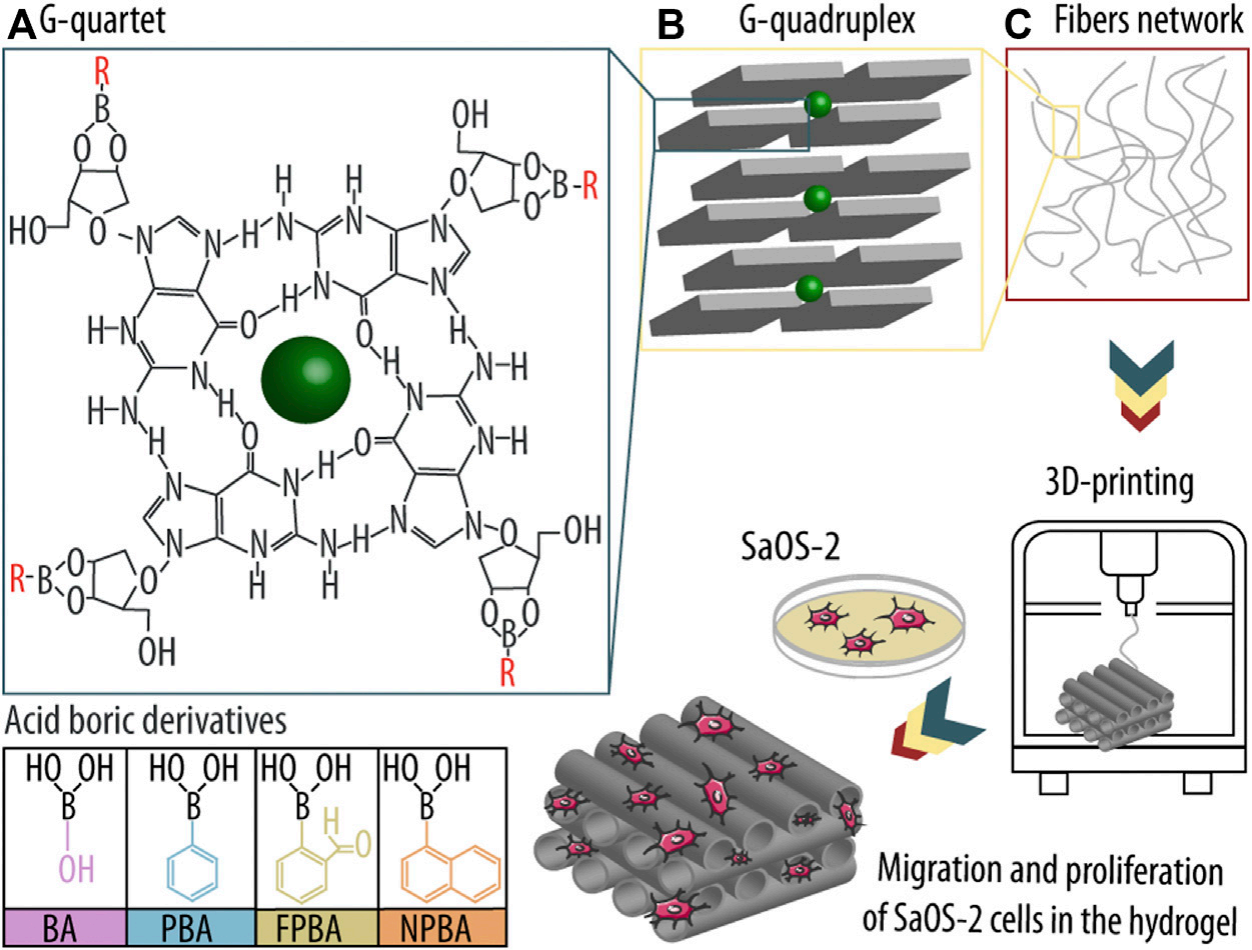
Guanosine (Guo)-based hydrogels formation using boric acid derivatives (dBAs) and subsequent three-dimensional (3D) printing and seeding of osteogenic sarcoma cells (SaOS-2). **(A)** First, Guo, dBAs, and potassium ions (K^+^) form an ordered G-quartet structure. **(B)** Four G-quartets interact and form a higher structure known as G-quadruplex, and ultimately **(C)** nanofibers, which build the hydrogel network. The assembly is then printed in a 3D printer, and SaOS-2 cells are seeded onto the scaffold, allowing them to migrate into the matrix and proliferate in the hydrogel system.

In particular, guanosine (Guo) and its derivatives such as guanosine 5′-monophosphate (GMP) have gained increasing attention in supramolecular chemistry and nanobiotechnology over recent years due to their unique self-assembly properties (Adhikari et al., 2014; Bhattacharyya et al., 2018). Guo exhibits several hydrogen donor and acceptor groups and is thus capable of forming ordered structures that can result under the correct conditions in dynamic hydrogels. For that, four Guo molecules form first a planar square arrangement called G-quartet *via* Hoogsteen base-pairing and by the coordination of a central monovalent cation (e.g., potassium ion; K+) by a carbonyl oxygen from each Guo monomer (Scheme 1A). Then, these G-quartets associate into a right-handed helical structure called G-quadruplex by π-π stacking and rotation by 30° (Scheme 1B), and by sharing the central metal ion (Carducci et al., 2018). Such G-quadruplex structures form a nanofibrous network (Scheme 1C) that is able to entrap large amounts of water, enabling this self-supporting structure to provide a biomimetic environment suitable for cell survival and differentiation. Thus, Guo-based hydrogels may be used in a variety of tissue regeneration or 3D printing applications due to their physicochemical properties such as i) biocompatibility, ii) biodegradability, iii) reversibility, and iv) tunability due to its non-covalent interactions (Do et al., 2015; Dong et al., 2015). However, classical Guo-based hydrogels have only a short-term stability, limiting their current use in tissue engineering (Rizwan et al., 2017; Yoneda et al., 2021).

To overcome this limitation, several strategies have been performed to improve the stability of Guo-based hydrogels and thus their applicability for tissue engineering. In particular, previous studies have shown that the incorporation of boric acids derivatives (dBAs) improved hydrogel stability *via* the formation of cis-1,2-diols between the Guo ribose ring with B(OH)_3_ or B(OH)_4_^−^. Especially in co-presence of potassium ions (K^+^), stable Guo/boric acid (BA) (Guo-BA) hydrogels could be formed (Adhikari et al., 2014). For example, Peters et al. (2014) detailed the formulation of stable Guo-BA hydrogels where the optimal combination of BA and K^+^ was the key determinant for the formation and stability of the hydrogel network. Over recent years, several studies confirmed the improved stability and long-term of Guo-BA hydrogels compared to other Guo-based formulations (Peters et al., 2014; Peters et al., 2015; Peters et al., 2016; Plank and Davis, 2016; Biswas et al., 2018a), and thus, Guo-BA hydrogels are currently considered the gold standard of Guo-based hydrogels (Bhattacharyya et al., 2017; Biswas et al., 2018b; Li et al., 2019; Qiao et al., 2020).

Combination of such hydrogels with 3D printing technology could provide customized scaffolds with a highly hydrated and controllable macro- and microporous (Wang et al., 2020) for optimal cell survival and differentiation due to its biomimetic properties. However, such biomaterials need exceptional good printability and self-healing properties to be able to form and maintain the printed structure and grant sufficient stability. For example, Biswas et al. (2018b) studied the properties of Guo-dBA (GB) hydrogels using phenyl boronic acid (PBA). The authors demonstrated that the dynamic nature of the boronic acid-boronate ester equilibrium produced injectable, self-healing and thixotropic properties for 3D printing. However, despite recent advances, various challenges remain, the most of which are connected to the physical properties of the 3D printed hydrogels and the functionality (e.g., proliferation, migration, and differentiation) of the embedded cells (Malda et al., 2013).

The dynamic behaviour of supramolecular hydrogels allows them to readily adapt to tissue defect sites upon injection and to subsequently support cell proliferation, migration, and differentiation. However, the very same dynamic properties typically also infer reduced mechanical strength. As a result, the incorporation of orthogonal non-covalent interactions (e.g., π-π stacks between additional aromatic rings) has been proposed (Criado-Gonzalez et al., 2020) to improve the mechanical properties of the self-assembled hydrogel without losing its tunability and self-healing capacity. Based on these premises, we designed and synthesized GB hydrogels by combining Guo, K^+^ ions, and four distinct dBAs (BA, PBA, 2-formilphenylboronic acid (FPBA) (Qiao et al., 2020; Li et al., 2022), and 2-naphtylboronic acid (NPBA) (Bhattacharyya et al., 2017)) since they were used in previous studies (Peters et al., 2014; Li et al., 2017; Biswas et al., 2018a; Ghosh et al., 2020; Qiao et al., 2020). We hypothesized that using dBAs containing aromatic rings will enhance G-quadruplex formation and, as a result, improve both hydrogel stability and printability due to the added π-π stack interactions. In detail, we i) tested different GB hydrogel compositions including four different dBAs, ii) characterized their hydrogel forming capacity and 3D printing properties, and iii) and evaluated their capacity to support viability and migration, of osteogenic sarcoma cells (SaOS-2) seeded onto the printed hydrogels.

## 2 Materials and methods

### 2.1 Materials

Guo, BA, PBA, FPBA, NPBA, phosphate-buffered saline (PBS), research grade fetal bovine serum (FBS), sodium pyruvate, penicillin-streptomycin (Pen-Strep; P/S), glutaraldehyde, hexamethyldisilazane (HDMS), bovine serum albumin (BSA), paraformaldehyde (PFA), Triton X-100, bovine serum albumin, Phalloidin-Atto 488 and DAPI ready-made solutions were all purchased from Sigma-Aldrich (Saint Louis, MO, United States).

Gibco™ Trypsin-EDTA (0.25%), phenol red, McCoy’s 5A (Modified) Medium, sterile syringe filters (polyethersulfone, 0.2 µm, 25 mm), Invitrogen™ CyQUANT™ LDH Cytotoxicity Assay and CellTracker™ Deep Red were purchased from Thermo Fisher Scientific (Waltham, MA, United States).

SaOS-2 cell line was purchased from ATCC^®^ (Manassas, VA, United States).

Potassium hydroxide (KOH) pellets EMPLURA^®^ were purchase from Merck KGaA (Darmstadt, Germany).

All solutions were prepared using ultrapure water from a Milli-Q Advantage A10 water purification system (EMD Millipore, United States) with a resistance of ≥18 MΩ cm and a total organic carbon (TOC) of ≤4 ppb.

### 2.2 Optimization and assessment of hydrogel formation

Weighed amounts of Guo were added into 2 mL microcentrifuge tubes (Eppendorf AG, Hamburg, Germany) with distinct volumes of KOH (300 mM) and dBAs stock solutions (300 mM) brough to a final volume of 500 µL with Milli-Q water. For example, 12.75 mg of Guo were mixed with 66.6 µL of KOH, 66.6 µL dBA and 366.8 µL of Milli-Q water. The resulting solutions were then heated to 80°C until the solutions were transparent and subsequently cooled at room temperature (RT) for ∼30 min without disturbance to allow hydrogel formation. The gelation of the various compositions was assessed by a simple inversion test (i.e., turning the tubes upside down), and images of the obtained hydrogels were taken with a Nikon D3100 camera (Tokyo, Japan) for further analysis. Various concentrations of Guo, dBAs and KOH were tested and the most promising compositions were used for printability evaluation.

### 2.3 Semi-quantitative assessment of printability

#### 2.3.1 Visual inspection test

GB hydrogels for printability assessment were formed by weighing accurate amounts of Guo into a 20 mL glass vial and adding solutions of KOH, dBA and Milli-Q water to a final volume of 4 mL. Mixtures were heated up to 80°C with stirring till the solutions became transparent. Then, the mixtures were transferred into 3 mL syringes with a parafilm sealed tip, and cooled down to RT for ∼1 h. The hydrogel solutions were 3D printed using a BIO X™ bioprinter from Cellink (Gothenburg, Sweden) at 37°C with a standard 20G nozzle (Cellink, Gothenburg, Sweden) having an inner diameter of 0.58 mm. The printing parameters were as follows: nozzle temperature of 37°C, substrate table temperature of 25°C, and a printing speed of 2 mm s^−1^. The printed hydrogel was made up of six layers, with a scaffold area of 20 mm × 20 mm × 3 mm (length × width × height). All tested samples were coded as X_Y, where X and Y represent the millimolar concentration of Guo (from 20 to 120 mM) and dBA/KOH (from 10 to 70 mM), respectively. For the visual inspection assay, a 6-layer scaffold was printed.

#### 2.3.2 Filament collapse test

**FIGURE 1:**
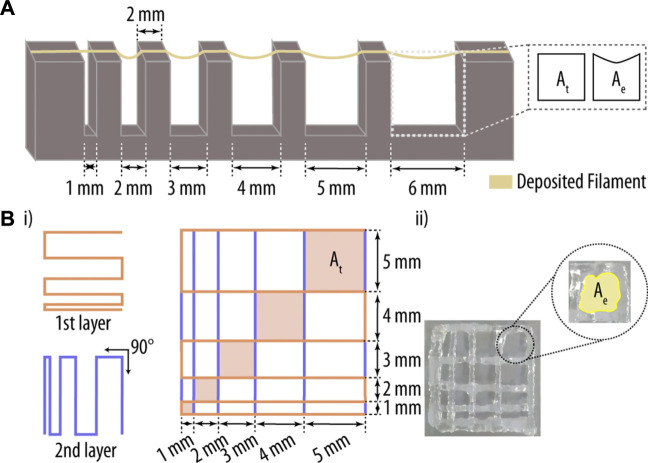
Printability assessment of hydrogel-based inks. **(A)** Three-dimensional (3D) printed platform used for the evaluation of the filament collapse area. While A_e_ represents the experimentally determined area, A_t_ corresponds to the theoretical area under the printed filament. **(B)** (i) Printing pattern used for the filament fusion test. Two filament layers were deposited along 0° and 90°, and the resulting areas between filaments (A_e_) were measured and compared to their theoretical counterparts (A_t_). Adapted with permission. [37] Copyright 2018, MDPI.

Filament fusion test has been described in detail elsewhere (Habib et al., 2018; Ribeiro et al., 2018; Habib and Khoda, 2019). Briefly, a platform consisting of seven pillars spaced in predefined intervals of 1, 2, 3, 4, 5, and 6 mm (Figure 1A) was designed in the CAD software SolidWorks (Dassault Systèmes, Waltham, MA, United States) and printed using a Ultimaker 2+ 3D printer (Utrecht, Netherlands) with polylactic acid (PLA) as base material. The five pillars in the middle have a dimension of 2 mm^3^ × 10 mm^3^ × 6 mm^3^ (W, H, D) and the corner-pillars are 5 mm^3^ × 10 mm^3^ × 6 mm^3^ (W, H, D).

Hydrogels were prepared as described in Section 2.3.1, and a single filament was printed onto the platform (Figure 1A). The deflected area of a hanging filament was analysed to determine the collapse area factor (C_f_; Eq. 1) of the printed hydrogel. In short, the nozzle-tip was placed 0.3 mm above the pillar surface, and the print path was extended 5 mm beyond the last pillar. Pictures were taken 1 min after filament deposition with a Nikon D3100 camera (Nikon corporation, Tokyo), and the area under the filament was analysed by ImageJ software (Schneider et al., 2012). The C_f_ is described as the percentage of the deflected area after filament suspension versus the theoretical area, as described in Eq. 1 (Ribeiro et al., 2018; Habib and Khoda, 2019):

$$C_{f}=\frac{A_{t}^{c}-A_{e}^{c}}{A_{t}^{c}}\times 100$$ (1)

where A^c^_e_ and A^c^_t_ are the experimental and theorical area, respectively, and low and high C_f_ values represent good versus bad filament stability.

#### 2.3.3 Filament fusion test

Extruded filaments of hydrogels with optimal gelation properties for 3D printing exhibit a consistent 3D width and a smooth surface, thereby providing the printed network with regular grids and square holes.

However, when the used hydrogel is under-gelated, the extruded filaments are partially liquid and the printed structures collapse along the *z*-axis, allowing the fusion of filament layers and resulting in more roundish holes. Thus, the grid and hole morphology of the printed structure is directly related to the fusion properties of the printed hydrogel. For the filament fusion test, a two-layer scaffold pattern with increasing filament to filament distance was designed, ranging from 1 to 5 mm in 1 mm increments (Figure 1B) (Habib et al., 2018; Ribeiro et al., 2018; Habib and Khoda, 2019). As before, pictures of the printed scaffolds were taken 1 min after printing using a Nikon D3100 camera, and the images analysed using ImageJ software (Schindelin et al., 2009). Parameters that are considered for this assay include: i) percentage of diffusion rate (Df_r_; rate of material spreading); ii) printability (P_r_; shape of the holes), and iii) angular deviation rate (D_a_; rate of non-rectangularity). Note, the angle deviation rate was only measured for the 5 mm^2^ × 5 mm^2^ square. These key printing parameters were determined using the following three equations (Habib et al., 2018; Ribeiro et al., 2018):

$${Df}_{r}=\frac{A_{t}^{f}-A_{e}^{f}}{A_{t}^{f}}\times 100$$ (2)

$$P_{r}=\frac{L^{2}}{{16A}_{e}^{f}}$$ (3)

$$D_{a}=\frac{\theta_{t}-\theta_{e}}{\theta_{t}}\times 100$$ (4)

Where A^f^_t_ and A^f^_e_ represent the theoretical and experimental area of the pore, respectively, L is the perimeter of the pore, while θ_t_ and θ_e_ are the theorical and experimental angles, respectively. Importantly, while low Df_r_ and D_a_ values indicate better printing qualities, a high P_r_ score is desired.

#### 2.3.4 Overall printability score

In order to select the best hydrogel concentrations, different scores were given to the samples depending on their behaviour during printability testing. The scoring for each test parameter is outlined in Table 1.

**TABLE 1:** Scoring table of the individual printing properties such as collapse area factor, diffusion rate, printability, and angle deviation rate.

Score	Collapse area factor (C_f_)	Diffusion rate (Df_r_)	Printability (P_r_)	Angle deviation rate (D_a_)
2	96%–100%	96%–100%	0.00–0.05	96%–100%
4	90%–95%	90%–95%	0.05–0.10	90%–95%
6	85%–90%	85%–90%	0.10–0.15	85%–90%
8	80%–85%	80%–85%	0.15–0.20	80%–85%
10	75%–80%	75%–80%	0.20–0.25	75%–80%
12	70%–75%	70%–75%	0.25–0.30	70%–75%
14	65%–70%	65%–70%	0.30–0.35	65%–70%
16	60%–65%	60%–65%	0.35–0.40	60%–65%
18	55%–60%	55%–60%	0.40–0.45	55%–60%
20	50%–55%	50%–55%	0.45–0.50	50%–55%
22	45%–50%	45%–50%	0.50–0.55	45%–50%
24	40%–45%	40%–45%	0.55–0.60	40%–45%
26	35%–40%	35%–40%	0.60–0.65	35%–40%
28	30%–35%	30%–35%	0.65–0.70	30%–35%
30	25%–30%	25%–30%	0.70–0.75	25%–30%
32	20%–25%	20%–25%	0.75–0.80	20%–25%
34	15%–20%	15%–20%	0.80–0.85	15%–20%
36	10%–15%	10%–15%	0.85–0.90	10%–15%
38	5%–10%	5%–10%	0.90–0.95	5%–10%
40	0%–5%	0%–5%	0.95–1.00	0%–5%

A maximum score of 40 was attributed for each of the four parameters. In particular, for filament collapse test, hydrogels with little or no deflection (i.e., C_f_ values between 0% and 5%) obtained the maximum score. For hydrogels without material spreading and/or with perfect right angle printing, Df_r_ and D_a_ are 0, and thus their score is maximum. For P_r_, values between 0.95 and 1 are considered best, corresponding to printed holes with a square shape. Finally, the total score of the different hydrogel formulations was normalized to 10.

### 2.4 Rheological studies

PLA molds (20 mm diameter) were designed using SolidWorks software and printed with a Sigma R19 3D printer (BCN3D technologies, Gavà, Barcelona). 2 mL of GB hydrogels (BA and NPBA) were transferred to the PLA molds and allowed to cool down at RT for ∼1 h without perturbation. Then, samples were gently removed from the molds and their rheological properties were studied using a Discovery HR-2 hybrid rheometer (TA Instruments, New Castle, DE, United States). Three different experiments were carried out in two distinct Peltier geometries: i) a strain sweep using a parallel stainless steel Peltier plate (20 mm diameter), ii) a dynamic step-strain sweep and iii) peak-hold assay with a cross-hatched Peltier plate (20 mm diameter).

The strain sweep was performed with an oscillation frequency of 1 Hz and a strain range from 0.01% to 100%. The dynamic strain sweep consisted of 10 alternate cycles of 100 s each with a strain ranging from 0.1% to 100% and a constant angular frequency of 10 rad s^−1^. The peak-hold assay had an initial step at a shear rate of 3.45 s^−1^ for 10 s, followed by a 60 s recovery step at a shear rate of 0.1 s^−1^. All experiments were repeated at least five times.

### 2.5 Morphological analysis of GB hydrogels

Two layers of the GB hydrogels (BA and NPBA) were printed using a Cellink BIO X™ bioprinter as detailed in Section 2.3.1. Then, samples were immersed in PBS containing 1% (w/v) glutaraldehyde for 1 h at RT. Next, scaffolds were washed three-times with PBS and dehydrated through a series of ethanol washes, each for 10 min (25%; 50%; 70%; 85%, 3 times, 95%, 3 times; and 100%, 3 times). Next, samples were immersed in HDMS for 10 min and dried at RT under vacuum. Finally, the GB hydrogels were coated with a layer of 10 nm of gold (Sputter coater AGB7340, Agar scientific, Stansted, United Kingdom) and analysed under a scanning electron microscope (SEM; Zeiss Merlin FE-SEM microscope, Carl Zeiss NTS GmbH, Germany). Five images were taken for each sample at distinct z-levels, a potential of 2 Kv, and a working distance of 5 mm. Additionally, 200 nanofibers were counted for each GB hydrogel using ImageJ (Schneider et al., 2012). Finally, two layers of Guo-BA and Guo-NPBA hydrogel were printed, as described in Section 2.3.1, and the scaffolds were stained with uranyl acetate for subsequent analysis using transmission electron microscopy (TEM; JEM-1010 microscope, JEOL, Tokyo, Japan). Five images at different positions were taken for each scaffold, and the respective diameter of the nanofibers was determined using ImageJ (Schneider et al., 2012).

### 2.6 Degradation and pH of GB hydrogels

Printed GB hydrogels were immersed in complete McCoy 5A medium and incubated at 37°C for 7 days, with changing media every 24 h. Images were taken at defined time points (0 h, 2 h, 4 h, 1 day, 3 days, 5 days, and 7 days) with a Nikon D3100 camera, and the printed GB hydrogel area was determined using ImageJ (Schneider et al., 2012). Additionally, the pH of the individual GB hydrogels was determined before printing using a SevenCompact Duo pH/Conductivity pH meter (Mettler Toledo, Mumbai, India). For each condition, three independent replicates were examined.

### 2.7 Cell experiments

#### 2.7.1 SaOS-2 cells culture

T175 cell culture flasks were used to plate 6 × 10^5^ cells cm^−2^ in McCoy’s 5A (Modified) medium containing 10% (v/v) FBS, 1% (v/v) P/S (10,000 U mL^−1^ and 10 mg mL^−1^, respectively), and sodium pyruvate (110 mg L^−1^). Subconfluent cells were detached by adding 4 mL of trypsin and incubation for 5 min at 37°C at 5% CO_2_. Cell suspensions were aspired and resuspended in complete fresh medium. Only cells up to passage 40 were used, and each of the three independent biological experiments included three technical replicates.

#### 2.7.2 SaOS-2 cells seeding on top of Guo-BA hydrogels

Weighed amounts of Guo were sterilized under UV for 20 min, while the KOH, the Milli-Q water, and the BA solutions were sterile filtered with a 0.2 µm polyethersulfone (PES) syringe filter. Guo-BA hydrogels were then prepared as described above (Section 2.3) under sterile conditions in a biological safety cabinet, and left to cool for 1 h in a 3 mL syringe. Magnets and nozzles were sterilized by immersion in ethanol (70%) for 30 min and subsequently washed three times with sterile PBS. Hydrogels were printed under sterile conditions using a Cellink BIO X™ bioprinter at 37°C, and a SaOS-2 cell suspension (50 µL, 10^5^ cells) was slowly and uniformly added onto the scaffolds to study cell migration into the hydrogels. Approximately 80%–90% of the scaffold surface was covered by the cells. The well plates were then incubated for 4 h at 37°C and 5% CO_2_, and 50 µL of fresh medium was added to each scaffold every 30 min for 4 h to prevent the cells from drying out and allowing for cell attachment. Next, 4.0 mL of fresh medium was added to each well, and the samples were incubated for the corresponding time period, with the cell medium being changed every other day.

#### 2.7.3 Cell viability and proliferation experiment

Viability and proliferation of SaOS-2 cells seeded onto 3D-printed Guo-BA hydrogels were assessed by detecting lactate dehydrogenase (LDH) activity in the culture medium after induced cell lysis using the commercially available Invitrogen™ CyQUANT™ LDH Cytotoxicity Assay (Thermo Fisher Scientific, Waltham, MA, United States). This assay is based on the conversion of lactate to pyruvate in the presence of LDH in parallel with a reduction of NAD^+^ to NADH. After incubation of the Guo-BA hydrogels with SaOS-2 cells for 1, 3, and 7 days, the scaffolds were washed three times with PBS to remove non- or loosely-attached cells. The washed scaffolds were then transferred to new wells and subsequently treated with 200 µL of 0.2% Triton X-100 in PBS to lyse the attached and incorporated cells and destroy the hydrogel scaffold. Then, 50 µL of the cell culture media were mixed with 50 µL assay reagent in a 96-well plate and incubated for 30 min at RT in the dark. Finally, absorbance at 490 and 680 nm was recorded using a microplate spectrophotometer system (Infinite M Nano, TECAN, Switzerland). The determined LDH activity was then converted into number of cells using a calibration curve. For that, SaOS-2 cells were seeded onto tissue culture polystyrene (TCPs) in increasing cell number. After 4 h of incubation, cells were washed and lysed as described above, and a calibration curve recorded of seeded cells versus LDH activity. As controls, cells that were likewise seeded onto TCPs were used.

#### 2.7.4 Cell morphology

Cell morphology was determined at 24 h (37°C and 5% CO_2_). Cells were washed twice in PBS to remove loosely attached cells, followed by fixation in 4% PFA (in PBS) for 30 min, and three additional PBS washes. For staining, cells were permeabilized with T-PBS (0.1% Triton X-100 in PBS) for 20 min at RT, and hydrogels were immersed in a 1% BSA solution (in PBS) for 1 h to minimize non-specific binding of the dye. SaOS-2 Guo-BA hydrogels were submerged in a solution of phalloidin-Atto 488 (0.1 μg mL^−1^ in PBS) for 1 h at RT in the dark to stain actin filament followed by three washes with PBS. Then the hydrogels were again incubated in the dark for 5 min at RT in a solution of DAPI (20 μg mL^−1^ in PBS). After three additional washes with PBS, hydrogels were imaged using the Leica TCS SP8 confocal laser scanning microscope (CLSM) equipped with an argon laser. Excitation/emission wavelengths of 405/440-480, respectively, were used for DAPI detection, and 488/495-545 for phalloidin-Atto 488. A ×63 oil immersion objective was employed. Cells seeded onto TCPS were similarly fixed, stained, and imaged as controls.

#### 2.7.5 Cell migration experiment

Many biological processes such as tissue formation depend on cell migration. Thus an *in vitro* assay was established to be able to assess the migration behaviour of the seeded cells. When SaOS-2 cells were confluent, the cell solution was treated with 5 μM CellTracker™ Deep Red (Thermo Fisher Scientific, Waltham, MA, United States) in serum free media and incubated at 37°C for 30 min following the manufacturer’s recommendations. After incubation, cells were subcultured and seeded in top of Guo-BA hydrogels as described above (Section 2.7.2). Cells performance labelled with Deep Red tracking was monitored up to 7 days using a CLSM (Leica Microsystems GmbH) equipped with an argon laser with a ×10 objective where excitation/emission wavelengths were set to 630–660 nm.

### 2.8 Statistical analysis

All data are presented as mean ± standard deviation and a non-parametric Mann-Whitney test was used to determine the statistical significance of differences in SPSS (IBM, Armonk, NY, United States) with a confidence level of 95% (*α* = 0.05; *p ≤ 0.05, **p ≤ 0.01, ***p ≤ 0.001 and ****p ≤ 0.0001), as the underlying data were not normally distributed.

## 3 Results and discussion

Guo-based hydrogels have gained increasing popularity as inks for tissue engineering due to their i) self-healing capacity, ii) high water retention potential, iii) biocompatibility, and iv) capability to provide an environment suitable for cell proliferation and survival (Dong et al., 2015). However, a major limitation of these hydrogels is their relative short-term stability, which can lead to the loss of their printed structure. However, the incorporation of additional non-covalent interactions, such as π-π stacks between the aromatic rings of added components, into the hydrogel network may be a viable strategy to overcome this limitation (Hayes and Greenland, 2015; Jin et al., 2019; Criado-Gonzalez et al., 2020). Such additional interactions may further stabilize the self-assembled hydrogel and thus improve its long-term mechanical properties. Among the various candidates, the use of dBAs is considered highly promising due to the possible enhancement of the hydrogel formation due to the additional interactions between the cis-1,2-diols of the Guo ribose ring with R-B(OH)_2_. Thus, we focused on a Guo hydrogel formed by the combination of KOH and distinct dBAs such as BA, PBA, FPBA and NPBA and optimized their printability and suitability as inks.

### 3.1 Optimization and assessment of hydrogel formation

**FIGURE 2:**
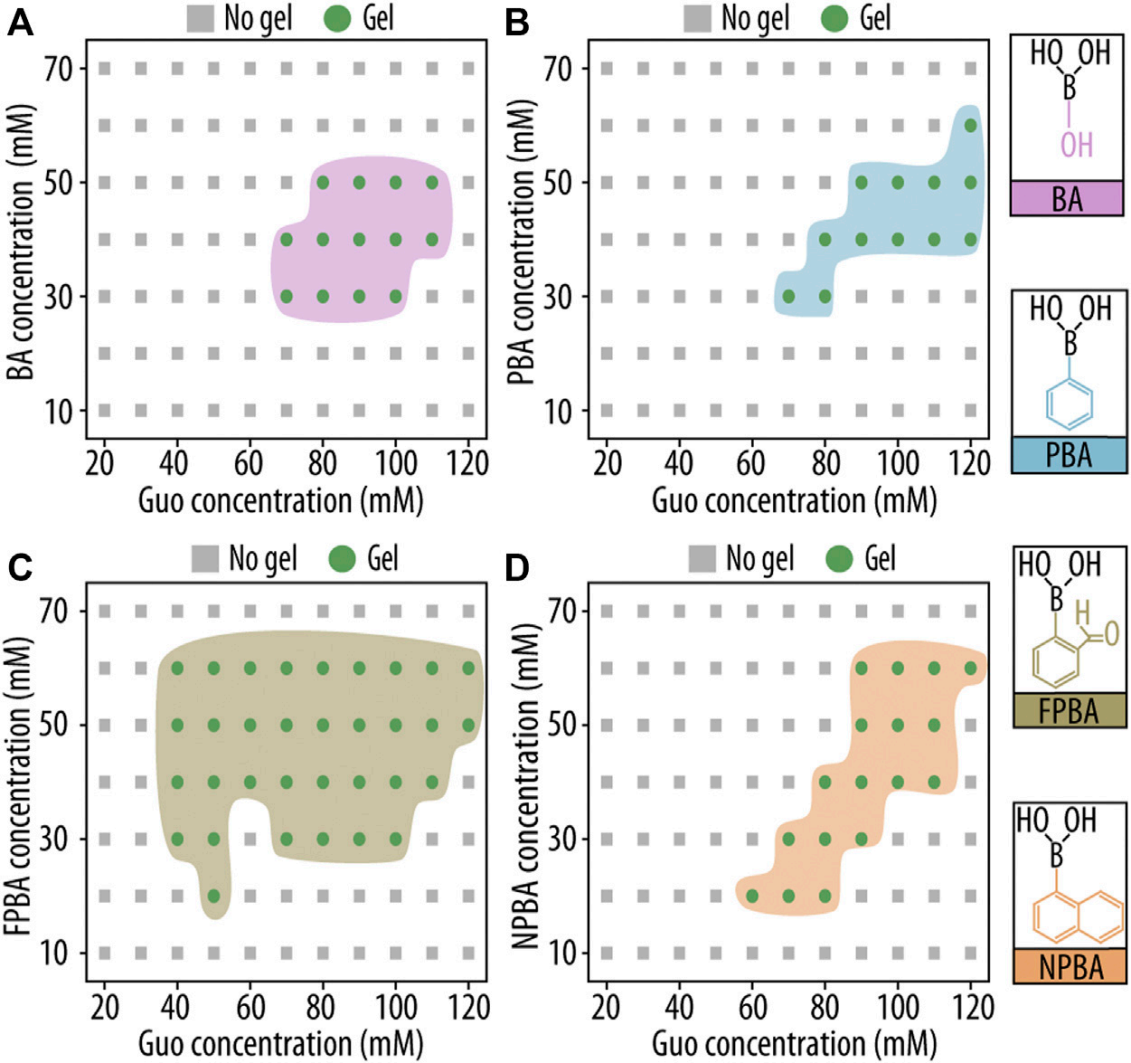
Gelation study of guanosine (Guo)-based hydrogels with boric acid derivatives (GB hydrogels) using a vial inversion test. Phase diagrams of gelation after 1 h at room temperature (RT) are shown as functions of Guo concentration and **(A)** boric acid (BA), **(B)** phenyl boronic acid (PBA), **(C)** 2-formilphenylboronic acid (FPBA), and **(D)** 2-naphtylboronic acid (NPBA) concentration.

First, we aimed to optimize the GB hydrogel compositions by evaluating the gel formation and assessing flow stability by inversion test (Figure 2). After 1 h at RT, hydrogel solutions were inspected visually, and the term “gel” was used for samples with a stable shape that did not move after inversion of the microcentrifuge tube. We tested different concentrations of Guo with KOH and BA, PBA, FPBA, or NPBA.

Hydrogels were formed in a wide range of conditions, but for all four dBAs, a minimal Guo concentration of approx. 40 mM was needed. Among the four dBAs studied, FPBA showed the highest number of gel-forming compositions. This may be explained by the extra aldehyde group present in FPBA providing additional interactions between distinct nanofibers. Critically, subsequent experiments were performed solely using formulations capable of forming stable gels without displacement during the inversion test.

### 3.2 Semi-quantitative assessment of printability

**FIGURE 3:**
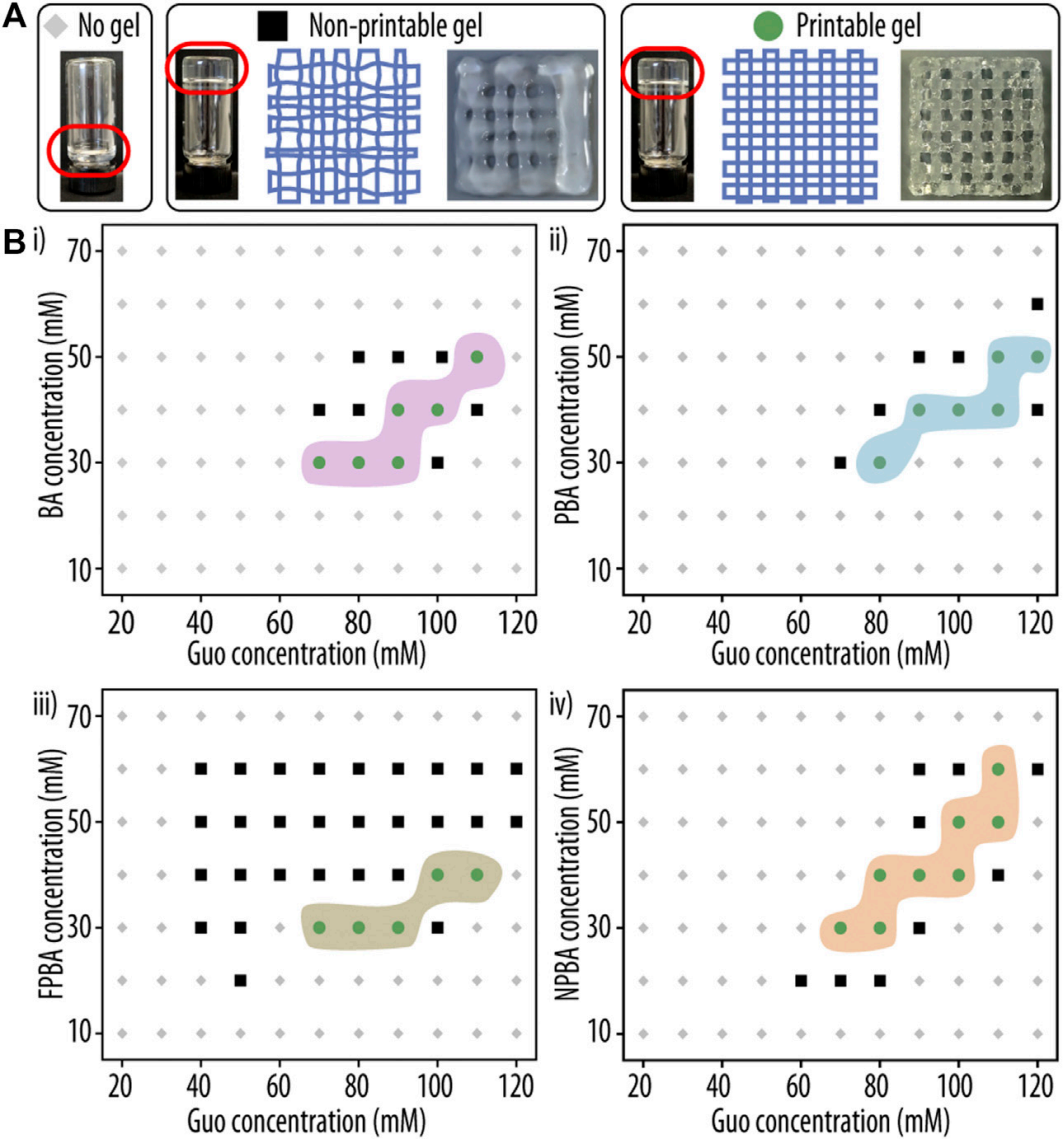
Printability assessment of guanosine (Guo)-based hydrogels with boric acid derivatives (GB hydrogels). **(A)** Representative images of hydrogel formulations yielding either no gel (left), a non-printable gel (middle), or a printable hydrogel (right) are shown. **(B)** Phase diagram of GB hydrogel gelation after 1 h at room temperature (RT) as a functions of Guo concentration and (i) boric acid (BA), (ii) phenyl boronic acid (PBA), (iii) 2-formilphenylboronic acid (FPBA) and (iv) 2-naphtylboronic acid (NPBA) concentration. Compositions resulting in three-dimensional (3D)-printable hydrogels are indicated as green circles, non-printable solutions as black squares.

New printing technologies and recent advances in hydrogel compositions, have resulted in materials that can provide a suitable environment for cell growth and differentiation while still displaying good structural properties. And by 3D printing, such biomaterials enable the fabrication of complex and patient-customized scaffolds (Yilmaz et al., 2021). First, we assessed the printability of the new inks by visual inspection of the printed scaffolds (Figure 3A), and only formulations resulting in discernible pores and layers and transparent gel structures were considered printable. This qualitative assessment gave us an initial impression of the shape fidelity of the various formulations, but was not intended to provide a score for the distinct composition.

Clear differences were observed between low and high Guo concentrations. At concentrations between 70 and 120 mM, printable hydrogels that did not cause nozzle jam were identified. On the other hand, at too low concentrations of Guo or dBAs/KOH, the hydrogels appeared under-gelated, and the printed scaffolds displayed filament spreading and filament fusion at the cross sites (Figure 3A). At too high Guo concentrations, the viscosity of the formed gels did not allow printing, resulting in nozzle jamming (Figure 3B). However, formulations identified as printable produced smooth filaments, and the printed grid structures showed well-defined squares and layers at the intersections. Noteworthy, no big differences could be observed between the different dBAs used (Figure 3B). This might be explained by the dBAs provided interactions being too few or too weak to significantly contribute to the stability of the supramolecular network.

Additionally, after cooling at RT and printing, opalescent gels were observed in all mixtures with FPBA and PBA, probably due to Guo precipitation and indicating the presence of Guo outside the G-quadruplex structure of the hydrogel network. Importantly, subsequent experiments were performed only with formulations that formed transparent gel structures (BA and NPBA) and passed this first round of evaluation, while FPBA and PBA were excluded.

**FIGURE 4:**
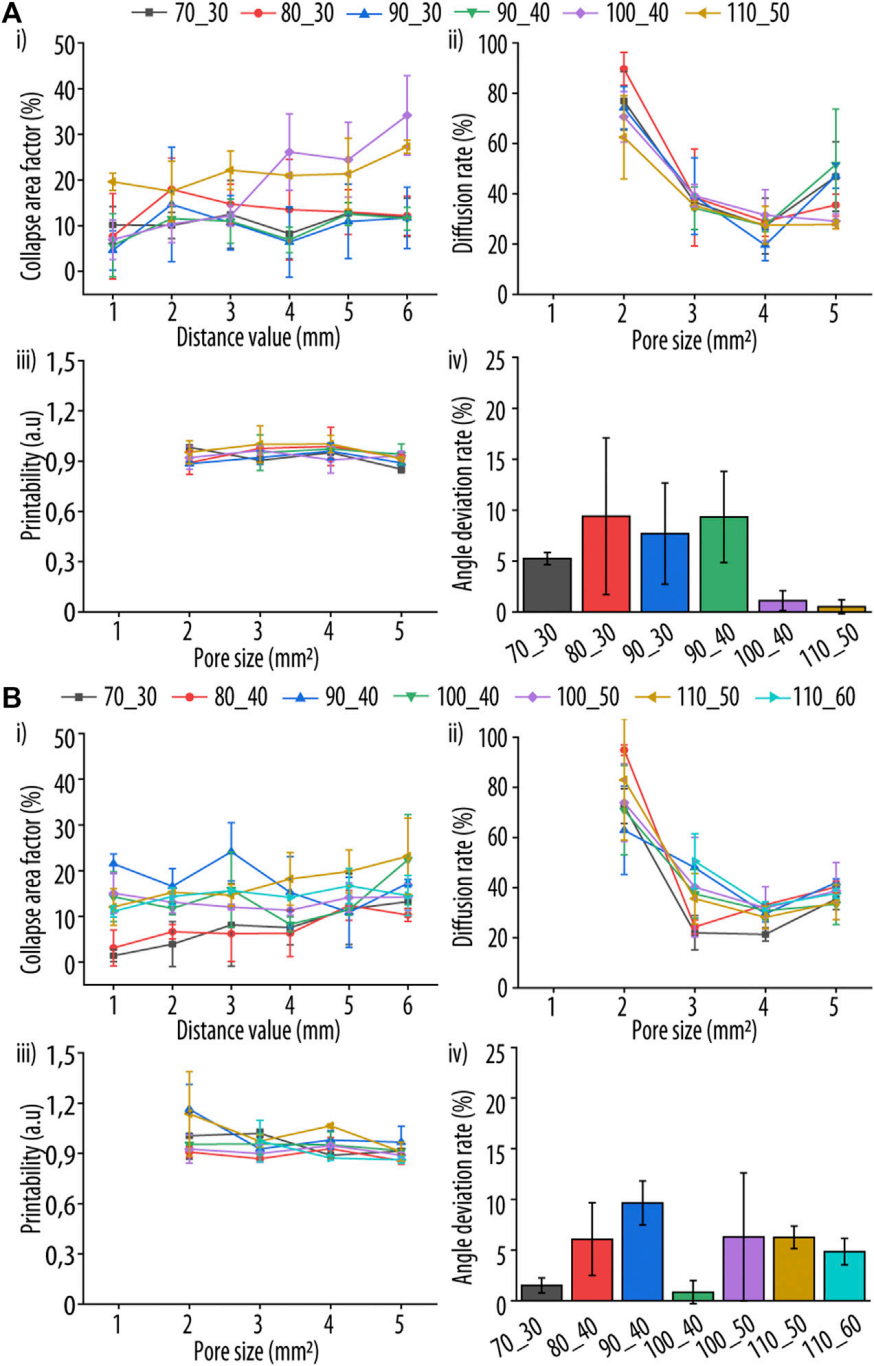
In-depth evaluation of two different ink parameters after printing to enable a comparative assessment of the tested guanosine (Guo)-acid boric derivatives (dBAs) (GB) formulation. Two dBAs were tested **(A)** boric acid (BA) and **(B)** 2-naphtylboronic acid (NPBA), and four different parameters evaluated: (i) collapse area factor, corresponding to the area of the hanging filament, (ii) diffusion rate, representing the material spreading on the printing surface, (iii) grid printability, corresponding to the perimeter of the printed grid squares, and (iv) angle deviation rate, reflecting the shape and rectangularity of the printed square holes, respectively. Samples are coded as X_Y, where X and Y represent the millimolar concentration of Guo and dBAs/KOH, respectively.

After selecting printable inks (from 70 to 120 mM for Guo and from 30 to 60 mM for dBAs/KOH) for both dBAs, we further continue testing the formulations in order to have the best composition for both Guo-BA and Guo-NPBA hydrogels for 3D printability. The individual hydrogel formulations were assessed and scored using two experimental set-ups and five distinct parameters (Ouyang et al., 2016; Habib et al., 2018; Ribeiro et al., 2018). All equations are described in the *Materials and methods* Section 2.3.2 and Section 2.3.3, and the qualitative assessments of the filament fusion and collapse tests are shown in Figure 4. Importantly, only minor or non-existent deformation was detected in the filament collapse test (Figure 4 i), indicating slow deformation rates in all selected hydrogel formulations. Noteworthy, such deformation rates are neglectable during printing as they happen in the timeframe of minutes.

Additionally, the C_f_ increased significantly with increasing Guo concentration. This could be explained by the increased density and consequently weight of such hydrogels, with the deformation being caused solely by gravity. This behaviour was found for all tested dBA, with no significant difference between them.

To estimate filament fusion, D_fr_, P_r_ and D_a_ were determined (Figure 4ii, iii, and iv). Two layers of hydrogels were printed onto a plastic surface, and only the upper layer was analysed to reduce the impact of the plastic-hydrogel interaction. No significant differences in D_f_ and P_r_ between the distinct Guo-BA and Guo-NPBA hydrogels formulations were observed and no fracture of filaments was produced during printing, indicating optimal hydrogel viscosity.

Angle printing accuracy is an important ink property as it reflects the behaviour of the ink during abrupt printing direction changes. Therefore, the top right square of each printed pattern was observed by joining the centre point of each corner and calculating the formed angle by ImageJ (Schneider et al., 2012) (Figure 1). For the angle deviation, Guo-BA and Guo-NPBA hydrogels showed distinct results with angle deviation rates lower than 12%. Additionally, for Guo-dBAs hydrogels tested at least one formulation with a low angle deviation rate could be identified.

**FIGURE 5:**
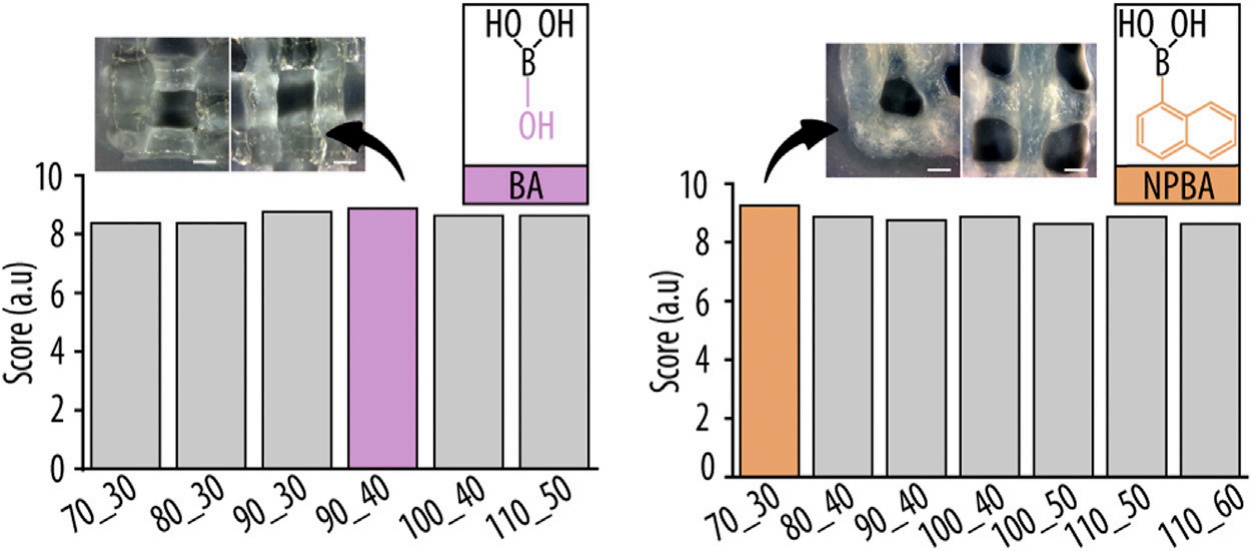
Summed and normalized printability scores of the individual hydrogel formulations obtained from filament fusion and collapse test. Inserts show representative images of the three dimensional (3D)-printed grid structures, highlighting corners (left) and filament cross-sections (right) for the best hydrogel compositions of boric acid (BA) and 2-naphtylboronic acid (NPBA), respectively. Scale bar 0.5 mm.

Based on these four hydrogel parameters, we devised a uniform scoring system (see Section 2.3.4) to enable a reproducible comparison of all hydrogel compositions. Figure 5 summarizes the final scores obtained after normalization from 1 to 10, and showing that the following dBAs formulations had the best printability and shape fidelity: 90_40 for Guo-BA hydrogel and 70_30 for Guo-NPBA hydrogel. The number before and after the underscore correspond to the used Guo and dBAs/KOH concentration (mM), respectively.

Many earlier studies relied on methods with few or no quantitative measures for the comparison of different hydrogels. Thus, we developed a scoring system that allowed us to compare hydrogel formulations in a reproducible manner and provided us with a semi-quantitative evaluation that allowed us to rank the individual hydrogels, something impossible to do based on a simple visual inspection of the filament or printed structure.

### 3.3 Guo-based hydrogels characterization

**FIGURE 6:**
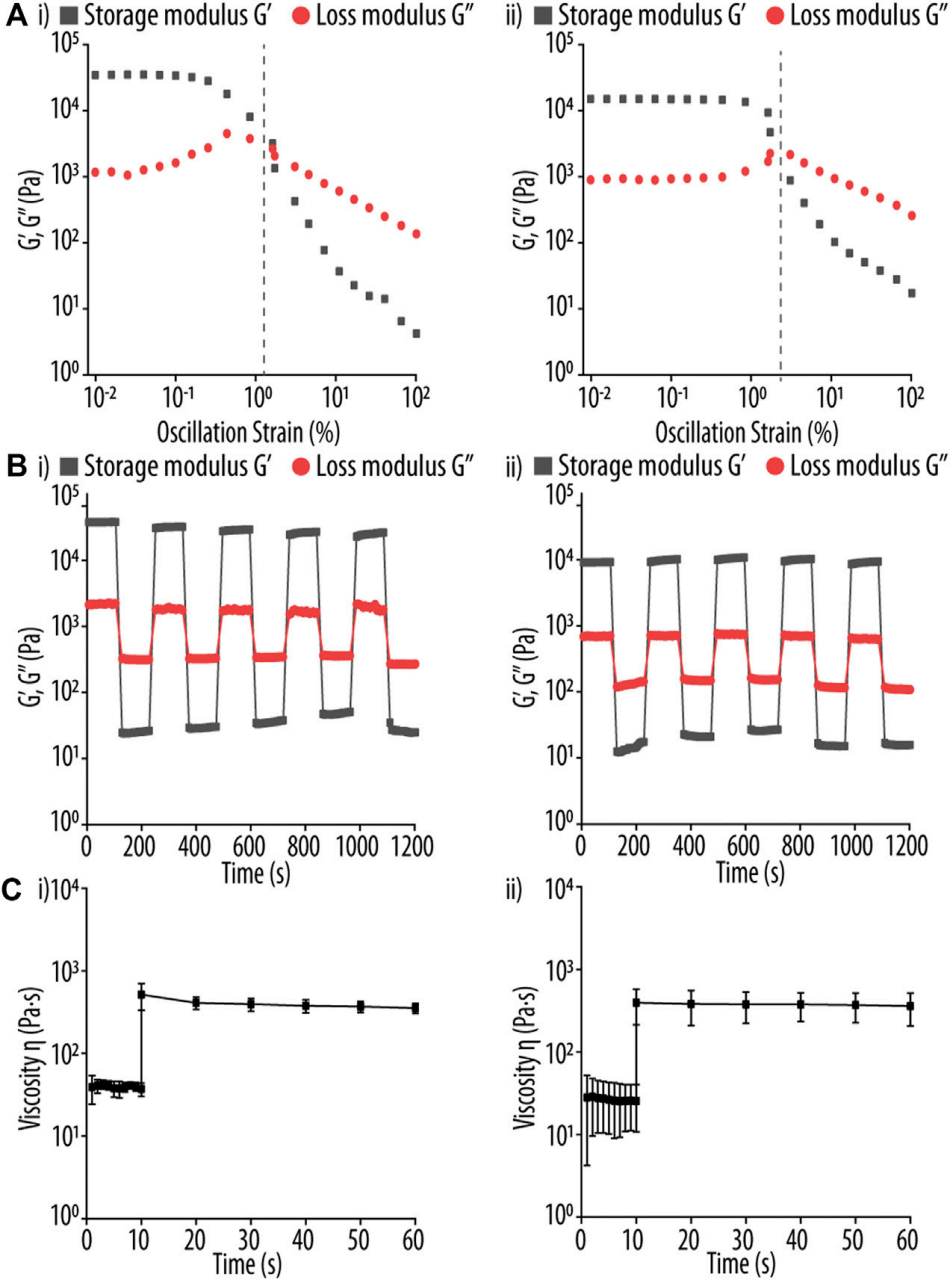
**(A)** Strain sweep test of guanosine (Guo)-based hydrogels using **(I)** boric acid (BA) and (ii) 2-naphtylboronic acid (NPBA). Storage modulus (G′, black square) and loss modulus (G″, red circle) were determined using a strain range of 0.01%–100% and a fixed oscillation frequency of 1 Hz. The respective turning point of both Guo-BA and Guo-NPBA hydrogels formulations in indicated by a vertical gray dashed line. **(B)** Rheological test of Guo-based hydrogels using (i) BA, and (ii) NPBA at 37°C. The G′ and G″ of the hydrogels are shown using a strain amplitude of 0.1% and 100% for the recovery and shear step, respectively, and a fixed angular frequency of 10 rad s^−1^. Each interval was constant at 100 s. **(C)** Rheological test of Guo-based hydrogels using (i) BA and (ii) NPBA at 37°C. A shear stress of 3.45 s^−1^ was applied for 10 s, mimicking the conditions during three dimensional (3D)-printing, and then the hydrogels were allowed to recover for up to 50 s at 0.1 s^−1^.

To study the mechanical properties of the 2 GB hydrogels selected in the previous section, we performed rheological measurements by monitoring the storage modulus (G′) and loss modulus (G″) at different test conditions such as strain sweep, dynamic step-strain sweep, and peak hold assay. Using strain amplitudes ranging from 0.01% to 100% at an oscillation frequency of 1 Hz, we determined the elastic nature of the hydrogels (Figure 6A). For both formulations, the G′ was higher than G″, but at certain strain amplitudes (1.6, 1.7 for BA and NPBA respectively) a turning point was observed. This region is known as the linear viscoelastic region (LVE), and it is used to identify the maximum strain value at which the gel maintains its elastic properties (Jungst et al., 2016; Rotaru et al., 2017), as amplitudes beyond that presumably lead to the breakdown of intermolecular or π-π stacking interactions. Therefore, we applied a strain of 0.1% in subsequent experiments to ensure an elastic nature of the hydrogels.

Next, we studied the self-healing properties and recovery times of the hydrogels. To do so, the strain was first kept at 0.1% for 100 s, and then raised to 100% in one step for an extra 100 s (Figure 6B) before returning to 0.1%. This procedure was repeated for five times for both the shear and recovery steps, and the results showed an elastic behaviour (i.e., G′ > G″) of the hydrogel at 0.1% strain, but the viscoelastic properties were lost (i.e., G′ < G″) at 100%. Importantly, after returning to a strain of 0.1%, both hydrogel formulations restored their initial characteristics within ∼50 s, by recovering ≥95% of their initial viscosity, making these two formulations excellent candidates for 3D printing. Moreover, after 100 s of recovery at 0.1% strain, the initial viscosity was fully recovered, indicating that the recovery time of the tested hydrogels is ∼100 s. This may be attributed to the dynamic non-covalent interactions in Guo-based hydrogels, such as hydrogen bonds, ionic interactions, or π-π stacking, which can easily be re-established. After a stress stimulus, such supramolecular interactions are able to reform the hydrogel network, and thus implement the hydrogels with self-healing properties. Thus, the two tested formulations presented outstanding thixotropic qualities, which are required for optimal injectability during printing.

Noteworthy, minor differences were noticed between both dBAs. G′ value for Guo-BA and Guo-NPBA hydrogels remained constant over all cycles. These results suggest that both hydrogel formulations exhibit a more solid-like behaviour and thus retain their elastic properties better when subjected to continuous deformation cycles (Tang et al., 2018; Thakur et al., 2019; Ghosh et al., 2021).

Furthermore, a peak-hold test was performed by applying a shearing stress equivalent to the shear rate in 3D printing (3.45 s^−1^), followed by a recovery step at 0.1 s^−1^. This test determines the amount of time necessary between printing different layers. The faster the gel viscosity recovers; the less time is required to print two non-merging filaments.

As evident from Figure 6C, the higher shear rate causes a decrease in viscosity but which stabilizes after approx. 3 s in all dBAs hydrogel samples. Then, under the low shear stress (recovery step), the viscosity of the system stabilizes within ∼1 s until the end of the experiment. Thus, this experiment clearly shows that the first layer of the printed hydrogel has sufficient time to stabilize before the second layer is printed, thus ensuring that the two filaments will not merge.

**FIGURE 7:**
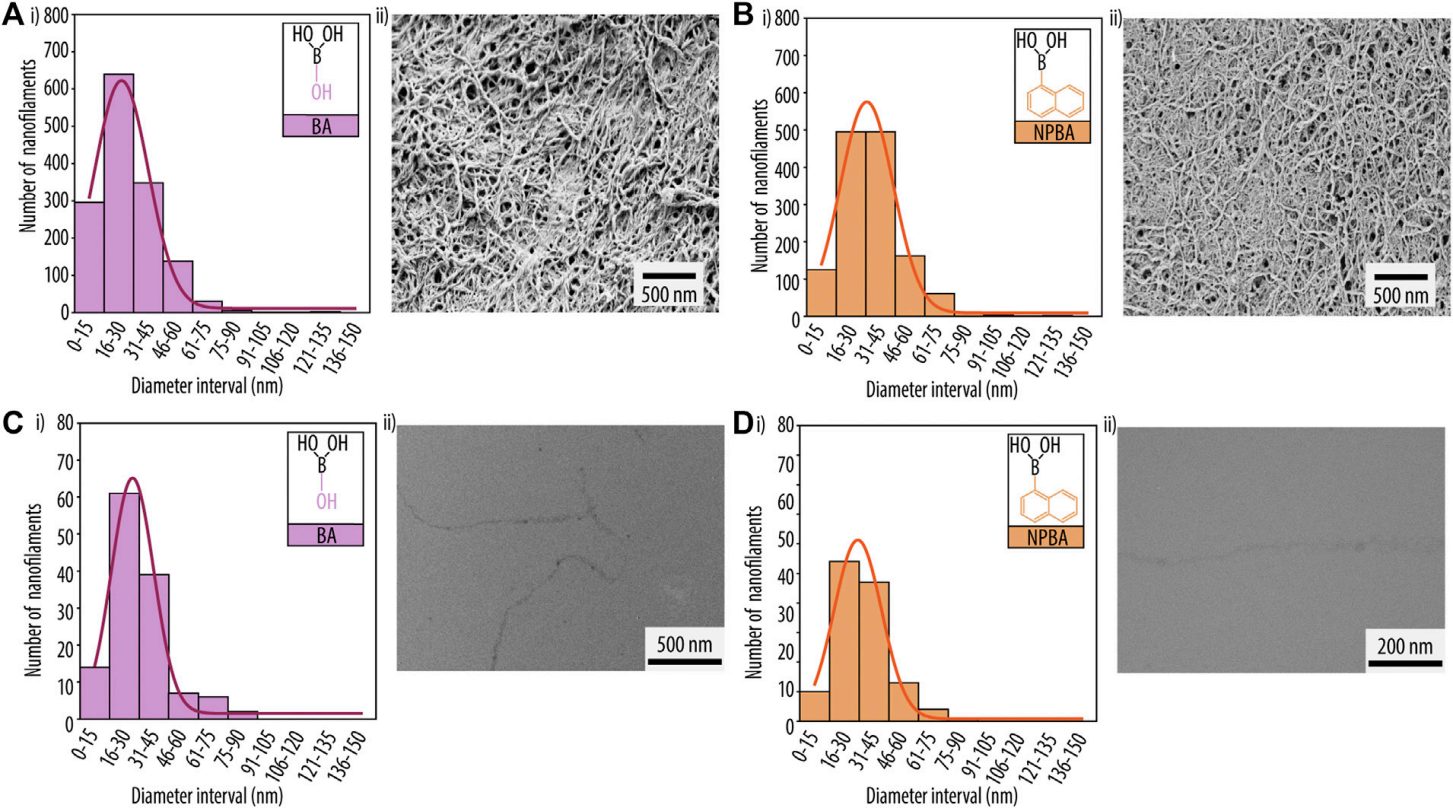
Scanning electron microscopy (SEM) analysis of printed hydrogels for guanosine (Guo)-based hydrogels using **(A)** boric acid (BA) and **(B)** 2-naphtylboronic acid (NPBA). (i) Diameter distribution histograms of the nanofibrillar network and (ii) SEM images. Transmission electron microscopy (TEM) analysis for **(C)** Guo-BA hydrogels and **(D)** Guo-NPBA hydrogels. (i) Diameter distribution histograms of the nanofibrillar network and (ii) TEM image.

Both hydrogel formulations resulted in good self-healing properties and a quick recovery period, confirming also the dominating non-covalent interactions (e.g., hydrogen bonds and π-π stacking) in such hydrogels. Notably, as favourable thixotropic properties indicate good injectability, the studied hydrogel compositions appear perfectly suited for 3D printing. Furthermore, we characterized the macroscopic morphology of the printed hydrogels by SEM. Figure 7 displays representative and well-defined nanofibrillar 3D networks for both printed hydrogels, with most nanofilaments ranging in diameter from 21 to 40 nm. In particular, a mean diameter of 27.8 ± 14.8 and 33.5 ± 15.4 nm was determined for BA and NPBA-based hydrogels, respectively, and their size distributions showed a full width at half maximum (FWHM) of 32.1 and 31.7 nm. Additionally, TEM images were analysed for both Guo-based hydrogels, confirming the SEM results, with a mean diameter of 23.8 ± 15.0 nm and 29.6 ± 13.6 nm for Guo-BA and Guo-NPBA hydrogels, respectively.

**FIGURE 8:**
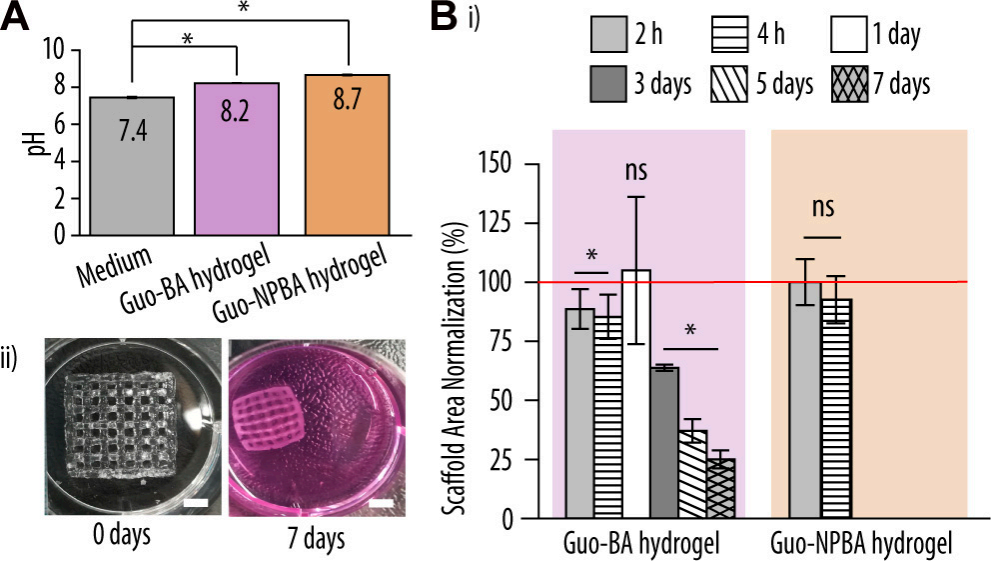
Biocompatibility evaluation of the printed hydrogel scaffolds. **(A)** pH values of the printed guanosine (Guo)-based hydrogels using boric acid (BA) and 2-naphtylboronic acid (NPBA) hydrogels vs. complete McCoy 5A medium. **(B)** (i) Normalized scaffold areas of printed Guo-BA and Guo-NPBA hydrogels after incubation in McCoy 5A medium at 37°C for up to 7 days. The red line represents 100% viability of the cells at 0 h (control). Difference testing was done versus control (ii) Representative image of guanosine-boric acid derivatives (GB) hydrogels after 0 and 7 days of immersion in complete McCoy 5A medium at 37°C and 5% CO_2_. Scale bar, 5 mm.

Overall, the selected Guo-dBAs based hydrogel formulations showed good printability properties. However, as we aimed to use these hydrogels as inks, we next evaluated their capacity to ensure cell survival. To do so, we first measured the pH of all formulations to confirm a suitable environment for cell growth (Figure 8A). While the pH of the Guo-BA hydrogel was 8.2 and was thus just slightly above the physiological pH of 7.45, the pH of the Guo-NPBA hydrogel was moderately basic (pH 8.7) and hence likely unsuitable for cell proliferation (Desai et al., 2012; Hu et al., 2017; Gaohua et al., 2021).

To further examine the stability of the Guo-based hydrogels, the printed gels were incubated in complete McCoy 5A medium at 37°C and 5% CO_2_. Previous studies had demonstrated that the main drawback of Guo-based hydrogels is their short stability in medium, limiting their use in biological applications (Biswas et al., 2018b; Dessane et al., 2020). Thus, we had hypothesized that changes in the supramolecular structure of the hydrogel, e.g., by the addition of additional aromatic rings and thus increased π-π stacking, would improve the hydrogel assembly and stability (Way et al., 2014; Pieraccini et al., 2019). Figure 8Bi shows the normalized scaffold area of both Guo-based hydrogels at distinct incubation times. Notably, while the Guo-BA hydrogel was stable for at least 7 days, Guo-NPBA hydrogels broke or dissolved after ∼4 h. This suggest that despite being embedded in the hydrogel network, NPBA is still reacting with the medium constituents (i.e., ions, glucose) (Wu et al., 2020), thus leading to a destabilization of the hydrogel network and a degradation of the printed structure after 2–4 h in medium. BA on the other hand is less reactive, keeping the interactions with the medium at a minimum, and thus the printed scaffold survived at least 7 days.

Although the normalized area of the Guo-BA scaffold decreased significantly over time, the overall structure and porosity remained unchanged (Figure 8Bii). This contraction (shrinkage) might be explained by the loss of water from the hydrogel network due to a higher ionic strength or different pH in the surrounding (Ionov, 2014).

Unfortunately, these results contradict our initial hypothesis that adding aromatic rings to the hydrogel formulation improves the properties of the printed scaffolds. Increasing the number of potential π-π stacking molecules in the hydrogel network did not only not improve hydrogel stability but even resulted in less stable scaffolds when placed in commercial cell culture medium. This might be explained by steric hindrance imposed by adjacent groups, thereby impairing the formation of π-π stack interactions between the additionally added aromatic rings. Thus, further experiments were limited to the Guo-BA hydrogel formulation as it was the only scaffold that retained its shape to some extent for more than 7 days.

### 3.4 Cell experiments

**FIGURE 9:**
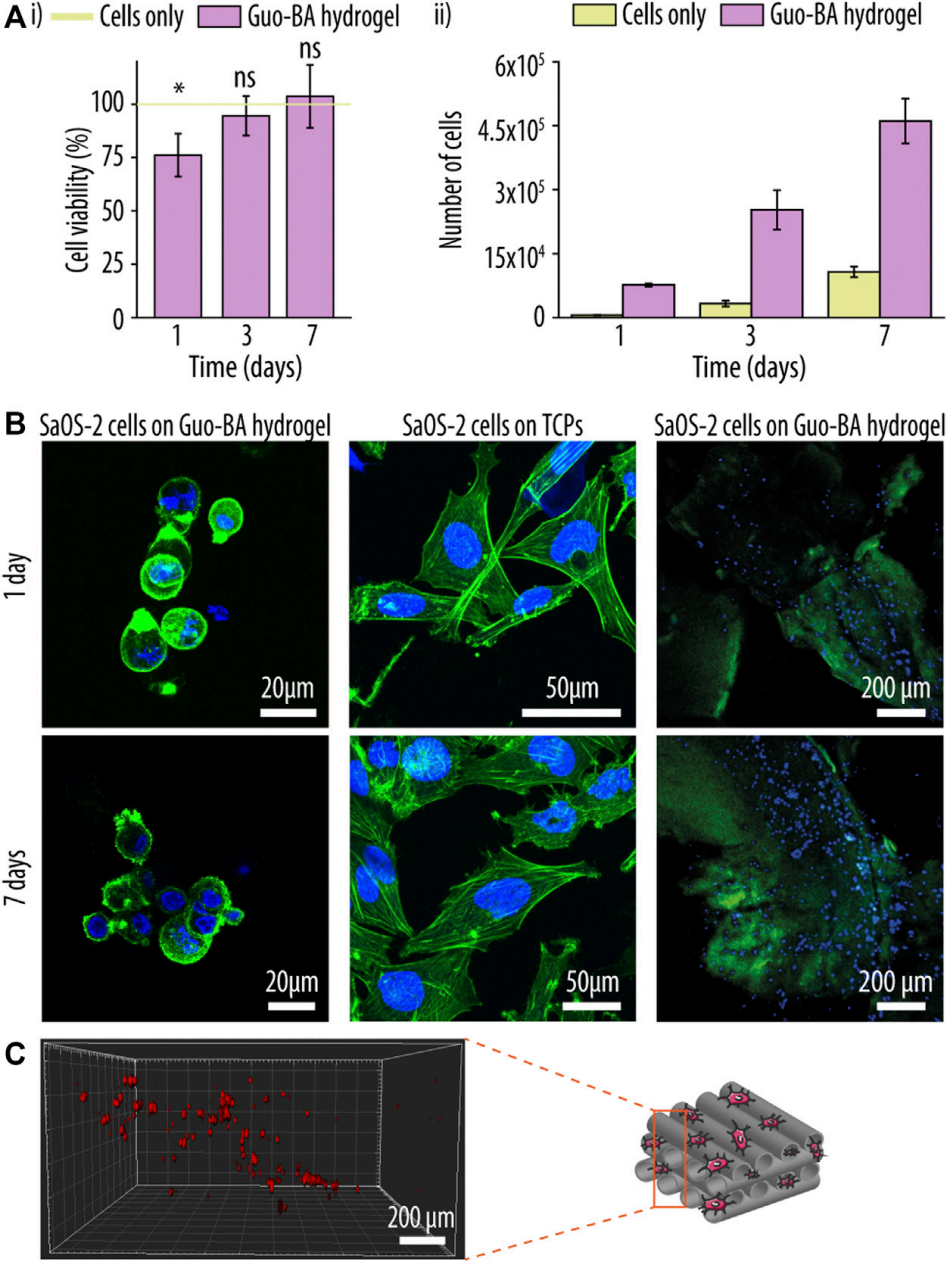
Post-printing cell functionalization of a guanosine (Guo)-based hydrogels with boric acid (BA) hydrogel. **(A)** Cell viability (i) and proliferation (ii) experiment of sarcoma osteogenic (SaOS-2) cells seeded onto the Guo-BA hydrogels up to 7 days. The green line represents 100% viability of the cells seeded on tissue culture polystyrene (TCPs) (control). Difference testing was done versus control. **(B)** Merged confocal laser scanning images of SaOS-2 cells morphology seeded on top of Guo-BA hydrogels, SaOS-2 cells morphology seeded on TCP and SaOS-2 cell distribution on Guo-BA hydrogels after 1 and 7 days of incubation. Actin filaments were stained with phalloidin-Atto 488 (green fluorescence signal) and nuclei with DAPI (blue fluorescence signal). **(C)** three dimensional (3D) representation of SaOS-2 cells migration through the Guo-BA hydrogel after 7 days. Cells were stained with CellTracker™ Deep Red reagent.

To evaluate potential applications of the developed Guo-BA hydrogel, a cell survival assay was performed using SaOS-2 cells that were seeded on top of the printed scaffold and analysed after 7 days of incubation. As shown in Figure 9Ai, the observed cell viability was at ∼78% after 1 day and ∼100% after 7 days, consistent with other studies that demonstrated Guo’s capacity to enable cell growth (Lanznaster et al., 2016; Massari et al., 2021). Additionally, the proliferation of the SaOS-2 cells was evaluated over a period of 7 days, showing a consistent rise in cell count. Notably, while cells initially proliferated faster when seeded onto the Guo-BA hydrogel, after 7 days the cell number was similar in both the TCPs well plate and the printed scaffold. This not only confirmed that the scaffolds were not cytotoxic, but also demonstrated that the hydrogel was capable to provide a suitable environment for SaOS-2 cell survival and proliferation (Do et al., 2015; Rotaru et al., 2017; Biswas et al., 2018b; Yoneda et al., 2021). In addition, cell morphology of SaOS-2 cells was evaluated by staining the actin filaments and the nuclei followed by CLSM. Regardless of the time point, the cells-maintained a roundish, non-differentiated morphology typical for suspension phase-like conditions (Figure 9B). Next, as evident by Z-stack analysis of confocal microscopy images and the presence of cells at various hydrogel levels, the seeded cells were capable of migrating and proliferating into the printed hydrogels, allowing for post-printing functionalization of such scaffolds with living cells (Figure 9C).

## 4 Conclusion

Guo-based hydrogels are of particular interest for biomedical purposes as they are not only biocompatible but also stimuli-responsive. In the past few years several systems based on Guo borate ester have been studied (Biswas et al., 2018b; Bhattacharyya et al., 2018; Li et al., 2019). Most recently, Qiao et al. (2020), for example, described a supramolecular hydrogel composed of Guo, FPBA, and KOH. However, prior research has not compared the impact of distinct dBAs on Guo-based hydrogel formation and their printability.

We hypothesized that by using dBAs containing aromatic rings would enhance G-quadruplex formation, and thus improve both hydrogel stability and printability due to the added π-π stack interactions. However, to our surprise, our results revealed that the additional aromatic rings did not only not improve the hydrogel properties but actually impaired them. We speculate that this behaviour is caused by steric hindrance imposed by adjacent groups, which interfere with the correct orientation of the aromatic groups needed for *bona fide* π-π stack interactions. Nevertheless, the here developed Guo-BA hydrogel showed good thixotropic properties and was able of sustaining cell survival, proliferation, and migration.

To conclude, our findings show that Guo-BA hydrogels represent promising scaffolds for tissue engineering that can be augmented with living cells, and we believe that our work represents a crucial stepping stone for the design of further functionalized Guo-based hydrogels.

## Data Availability

The raw data supporting the conclusion of this article will be made available by the authors, without undue reservation.
