# Supplementary material for: Optimization of guanosine-based hydrogels with boric acid derivatives for enhanced long-term stability and cell survival
Source: Front Bioeng Biotechnol. 2023 Mar 20;11:1147943. doi: 10.3389/fbioe.2023.1147943 (PMC10069680; doi:10.3389/fbioe.2023.1147943)
Supplement: Supplementary file 1 [file DataSheet1.docx]

Supplementary Material

Optimization of guanosine-based hydrogels with boric acid derivatives for enhanced long-term stability and cell survival

Maria Merino-Gómez, Maria Godoy-Gallardo^*^, Mathias Wendner, Miguel A. Mateos-Timoneda, F. Javier Gil, Roman A. Perez^*^

*** Correspondence:** Maria Godoy-Gallardo: [mgodoy@uic.es](mailto:mgodoy@uic.es); Roman A. Perez. rperezan@uic.es

**Table 1.** Scoring table of the individual printing properties such as collapse area factor, diffusion rate, printability, and angle deviation rate.

| **Score** | **Collapse area factor (C_f_)** | **Diffusion rate (Df_r_)** | **Printability (P_r_)** | **Angle deviation rate (D_a_)** |
| --- | --- | --- | --- | --- |
| **2** | 96 – 100% | 96 – 100% | 0.00 – 0.05 | 96 – 100% |
| **4** | 90 – 95% | 90 – 95% | 0.05 – 0.10 | 90 – 95% |
| **6** | 85 – 90% | 85 – 90% | 0.10 – 0.15 | 85 – 90% |
| **8** | 80 – 85% | 80 – 85% | 0.15 – 0.20 | 80 – 85% |
| **10** | 75 – 80% | 75 – 80% | 0.20 – 0.25 | 75 – 80% |
| **12** | 70 – 75% | 70 – 75% | 0.25 – 0.30 | 70 – 75% |
| **14** | 65 – 70% | 65 – 70% | 0.30 – 0.35 | 65 – 70% |
| **16** | 60 – 65% | 60 – 65% | 0.35 – 0.40 | 60 – 65% |
| **18** | 55 – 60% | 55 – 60% | 0.40 – 0.45 | 55 – 60% |
| **20** | 50 – 55% | 50 – 55% | 0.45 – 0.50 | 50 – 55% |
| **22** | 45 – 50% | 45 – 50% | 0.50 – 0.55 | 45 – 50% |
| **24** | 40 – 45% | 40 – 45% | 0.55 – 0.60 | 40 – 45% |
| **26** | 35 – 40% | 35 – 40% | 0.60 – 0.65 | 35 – 40% |
| **28** | 30 – 35% | 30 – 35% | 0.65 – 0.70 | 30 – 35% |
| **30** | 25 – 30% | 25 – 30% | 0.70 – 0.75 | 25 – 30% |
| **32** | 20 – 25% | 20 – 25% | 0.75 – 0.80 | 20 – 25% |
| **34** | 15 – 20% | 15 – 20% | 0.80 – 0.85 | 15 – 20% |
| **36** | 10 – 15% | 10 – 15% | 0.85 – 0.90 | 10 – 15% |
| **38** | 5 – 10% | 5 – 10% | 0.90 – 0.95 | 5 – 10% |
| **40** | 0 – 5% | 0 – 5% | 0.95 – 1.00 | 0 – 5% |

**
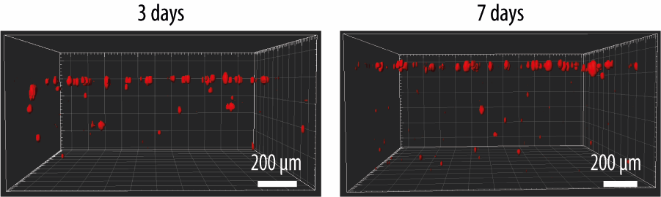
**

**Figure S1**. Three dimensional representation of SaOS-2 cells migration through the guanosine-boric acid hydrogel after 3 and 7 days. Cells were stained with CellTracker™ Deep Red reagent.
